# Atomic scale insight into the effects of Aluminum doped Sb_2_Te for phase change memory application

**DOI:** 10.1038/s41598-018-33421-y

**Published:** 2018-10-11

**Authors:** Yong Wang, Tianbo Wang, Yonghui Zheng, Guangyu Liu, Tao Li, Shilong Lv, Wenxiong Song, Sannian Song, Yan Cheng, Kun Ren, Zhitang Song

**Affiliations:** 1 State Key Laboratory of Functional Materials for Informatics, Laboratory of Nanotechnology, Shanghai Institute of Micro-System and Information Technology, Chinese Academy of Sciences, Shanghai, 200050 China; 2 Hangzhou Dianzi Univ, Coll Mat & Environm Engn, Hangzhou, Zhejiang 310018 China; 3 University of Chinese Academy of Sciences, Beijing, 100049 China; 4 Key Laboratory of Polar Materials and Devices, Ministry of Education, East China Normal University, Shanghai, 200062 China

## Abstract

To date, the unpleasant trade-off between crystallization speed and thermal stability for most phase change materials is detrimental to achieve phase change memory (PCM) with both features of high-speed and good-retention. However, it is proved that Al doping in Sb_2_Te, served as storage media in PCM, favors both a high writing speed (6 ns) and a good retention (103 °C), as well as a low power consumption. Judging by experimental and theoretical investigations, doped Al atoms prefer to replace Sb in Sb_2_Te lattice, strongly bonded with 6 Te atoms, to form a homogeneous phase. While in amorphous Al doped Sb_2_Te (AST), Al atoms are in tetrahedral environment, firmly bonded with four Sb/Te atoms. The strong bonding in Al centered tetrahedron in amorphous AST can obstruct the collective motion of Sb atoms near the matrix boundary, leading to the improvement in thermal stability and the confinement in grain size.

## Introduction

In the last decades, phase change memory (PCM) has become one of the most promising candidates to complement the existing memory hierarchy, with the performance and cost lying between that of NAND flash and DRAM^1–4^. In PCM, data encoding is accomplished by controlling the resistive states of phase change material through electrical pulses, where amorphous state (Reset state) is highly resistive, while crystalline state is highly conductive (Set state)^5^. The features of phase change materials, including thermal stability of glass state, crystallization speed, grain size, are the key factors that determine the performance of PCM, that is retention, writing speed, endurance, etc. Seeking for phase change materials that can lead to better performance is an abiding hot topic in PCM research. However, some aspects of PCM performance are statistically against each other, such as retention and writing speed. Luckily, material engineering based on the understanding of the molecular dynamic shed some light on making a breakthrough.

The introduction of GeTe-Sb_2_Te_3_ pseudobinary alloys by N. Yamada, *et al*., especially Ge_2_Sb_2_Te_5_ (GST), is one of the most success in the development of phase change materials, which served as the storage media in optic disc^6–9^. However, when apply GST to PCM, the mediocre performance of Set speed (~50 ns) and retention (85 °C, 10 years) has made it unsatisfactory for high speed/retention application^10^. Doped Sb_2_Te alloys is another materials family that often used as storage media in optic disc and PCM, e.g. Ag-In-Sb-Te (AIST). It is found that the crystallization of AIST alloys can be viewed as a sequential, collective motion of Sb atoms requiring neither bond breaking nor diffusion. Crystal growth can be speeded up by the lacked cavities and chemical alternation in Sb-rich Sb-Te alloys^11^. Owing to these facts, Sb_2_Te (ST) can be a promising candidate for achieving high-speed cache-level memory. Nevertheless, there are some critical shortcomings that prevent ST from its application in PCM: the poor thermal stability of the amorphous phase that limits the retention; the large grain size that endangers the endurance. Nowadays, various ST related materials have been tried, in the aim of exploring X-Sb-Te (X = dopant element) alloys with superior performance^12^.

In our previous work, the influence of some dopants on X-Sb-Te has been studied, such as Si^13^, N^14^, Zr^15^, Cr^16^, W^17^, Mo^18^, which can improve the Set speed to ~ 10 ns, generally. Other researchers have been focusing on the Si^19^, W^20^, Zn^21^, and Cu^22^ doping in ST, yet very few device performances have been reported. In addition, the influence of dopants on ST by both experimental and theoretical methods has rarely been studied.

The coordination number of Al can be six or four, determined by the type of orbits hybridization, that is, 3s^1^3p^3^3d^2^ or sp^3^. The flexibility of orbits geometry allows Al to undergo reversible tetrahedron-octahedron reconfigurations in phase change material during reversible phase transition, as reported by Xia *et al*., analogous to the movement of Ge in GST. Moreover, the strong bonding participated with Al, can enhance the rigidity of the amorphous atoms metrics, thus, leading to a high thermal stability of amorphous phase^23^.

In this work, Al doped ST (AST), more specifically Al_0.3_Sb_2_Te, is proposed for PCM application, with the aim of simultaneously improving the crystallization speed and enhancing the thermal stability, while causing no segregation. *Ab-initio* theoretical simulation is applied to give an insight view of the role Al dopant played in AST, that changes the reversible phase change behavior. The combination of experimental studies and theoretical calculations provides a novel way of designing and investigating advanced phase-change materials for high-speed and high retention PCM application.

## Results

**Figure 1:**
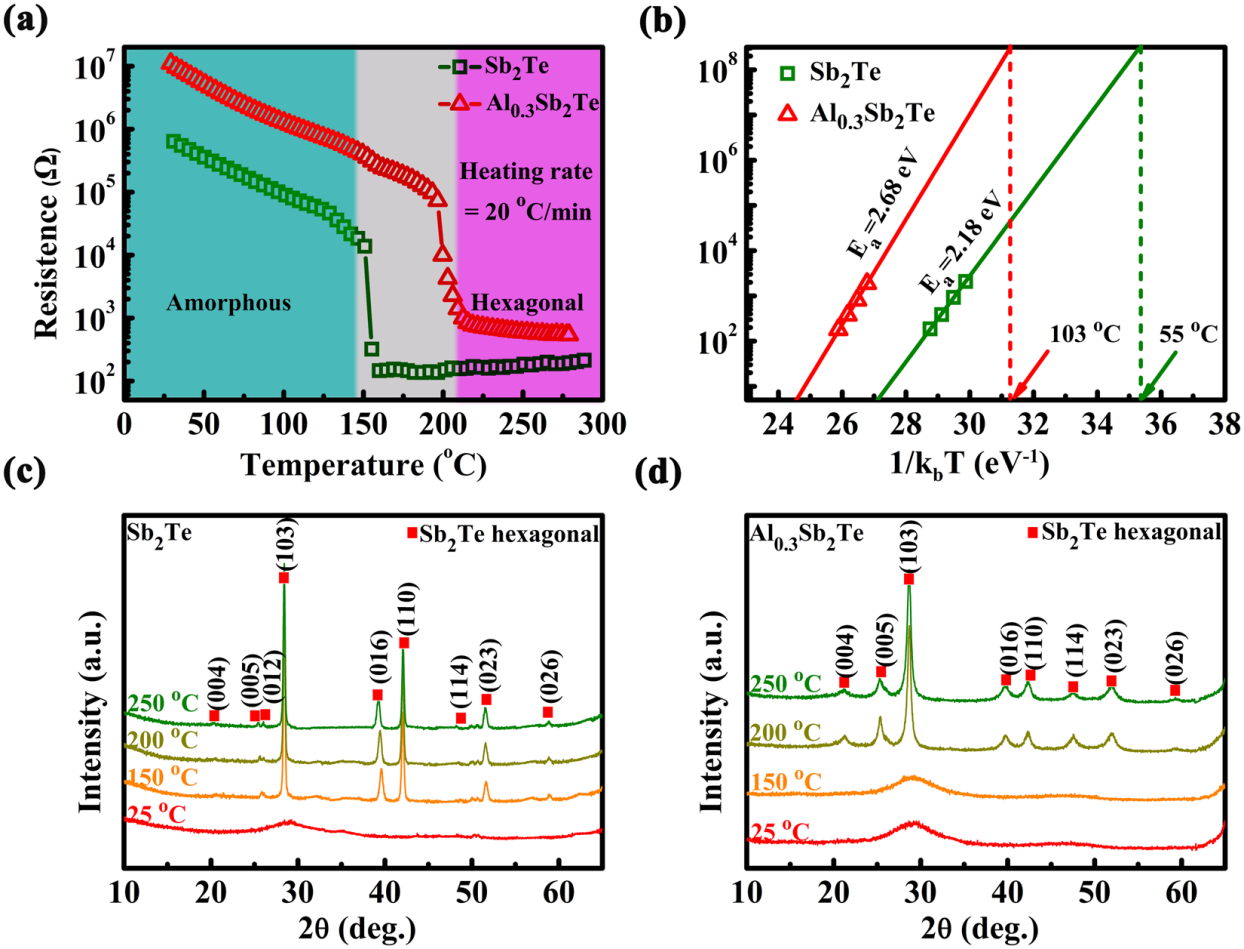
(**a**) The sheet resistance as a function of *in-situ* annealing temperature for the ST and AST films. (**b**) Plots of the failure time versus the reciprocal temperature (isothermal), showing temperatures for 10-year-dataretention from which the crystallization activation energy of ST and AST material is determined. (**c**,**d**) XRD curves of ST films and AST films at different annealed temperatures, where hexagonal lattice planes of Sb_2_Te can be identified as red dots.

*In-situ* resistance-temperature (*R-T*) and XRD measurements for ST and AST films are performed to study the thermal properties and to confirm the lattice structure of AST material. Fig. 1(a) shows sheet resistance (*R*_*s*_) of the as-deposited ST and AST films as a function of annealing temperature at a ramp rate of 20 °C/min, respectively. The as-deposited ST and AST films are confirmed to be amorphous with a high resistance. As temperature increases, the sheet resistance of both ST and AST decreases, presenting a semiconductor-like behavior. The point where the sheet resistance starts to decrease drastically is defined as the crystallization temperature (*T*_*c*_). *T*_*c*_ of AST is ~200 °C, higher than that of ST ~150 °C, implying the thermal stability has been improved by Al doping. Meanwhile, the resistance of amorphous and crystalline AST has been increased by Al doping, which will contribute to a higher efficiency of Joule heating, as well as a lower programming current^24^.

Figure 1(b) presents the data retention of ST and AST, predicted according to Arrhenius equation:

1 $${t}={\tau }\,\exp ({E}_{a}/{k}_{B}T)$$

where $\tau$, ${E}_{a}$, and ${k}_{B}$ are a proportional time constant, activation energy of crystallization, and Boltzmann’s constant, respectively. The failure time (*t*) is defined by the time when the resistance decreased to half of its initial value at the specific isothermal annealing temperature (*T*). According to eq. (1), ${E}_{a}$ and the 10-year data retention temperature (*T*_*d*_) can be estimated from the extrapolated fit line. Here, the ${E}_{a}$ of ST and AST films are ~2.18 eV and ~2.68 eV, respectively. The estimated *T*_*d*_ increases from ~55 °C (ST) to ~103 °C (AST) after Al doping, with the latter higher than that of GST (~85 °C), competent for consumer electronics application^25^.

**Figure 2:**
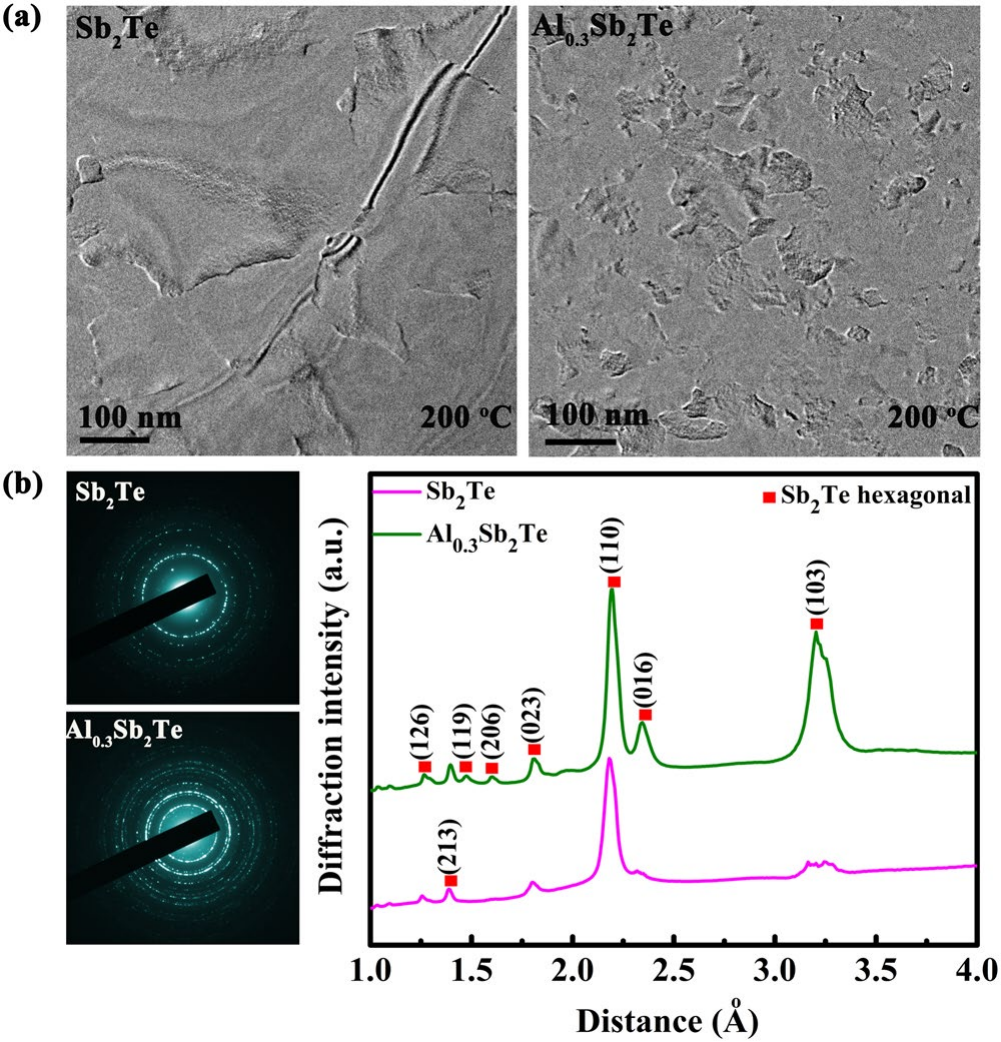
(**a**) Bright-field transmission electron microscopy images at 200 °C of ST and AST, respectively. (**b**) The corresponding selected area electron diffraction (SAED) patterns and the raw radically integrated diffraction curves of electronic diffraction intensity extracting from the respective SAED patterns.

Fig. 1(c,d) display the XRD patterns of ST and AST films annealed at different temperatures. The annealing process takes 2 min for each sample, under a N_2_ atmosphere. The as-deposited ST and AST films are amorphous since no diffraction peak appears in the curve. ST films has crystallized into a hexagonal phase since annealing at 150 °C, while AST keeps amorphous until annealing temperature reaches 200 °C. The diffraction peaks denoted by the red dots in Fig. 1(c,d) can both be indexed to hexagonal ST. No miscellaneous diffraction peak is detected in AST films which implies that incorporation of Al does not cause phase separation in crystalline AST. Compared to the diffraction results of ST, the diffraction peaks of the AST films at 28.5° (103), 39.1° (016), and 42.3° (110) look weaker in the intensity and larger in the full-width at half maximum (FWHM), that can be regarded as an evidence of smaller size of grains in AST, compared to that of ST, according to the Scherrer formula^26^. In phase change material with more confined grains, there will be less stress during reversible phase transition and higher uniformity of element distribution, which favor a higher device reliability and a greater potential for scaling down. The grain growth will be further studied by transmission electron microscopy (TEM) in Fig. 2.

TEM method is applied to have a direct observation of the distribution and size of the grains in the 200 °C annealed ST and AST films (~15 nm thick) as shown in Fig. 2. Sb_2_Te grains grow into a size of >200 nm, as shown in Fig. 2(a). In AST sample, the grain size has decreased remarkably after Al doping, showing a size of 50–100 nm. Smaller grains can provide more interfaces that will scatter the carriers when current flows through the device cell, resulting in a higher resistance in AST compared to that of ST. The increased resistivity favors the generation of Joule heat in AST, reducing the programming current. Thus, a high heating efficiency and a low power consumption can be achieved in AST based PCM. Figure 2(b) exhibits the corresponding selected area electron diffraction (SAED) rings and the raw radially integrated diffraction curves of electronic diffracting intensity (EDIC) extracting from the SAED patterns^27^. The SAED rings of ST films are more discontinued than that of AST films, denoting the larger grains in the former. By indexing EDIC, both ST and AST are belonging to hexagonal Sb_2_Te lattice. The absence of miscellaneous diffraction peak suggests a homogeneous AST material without segregation.

**Figure 3:**
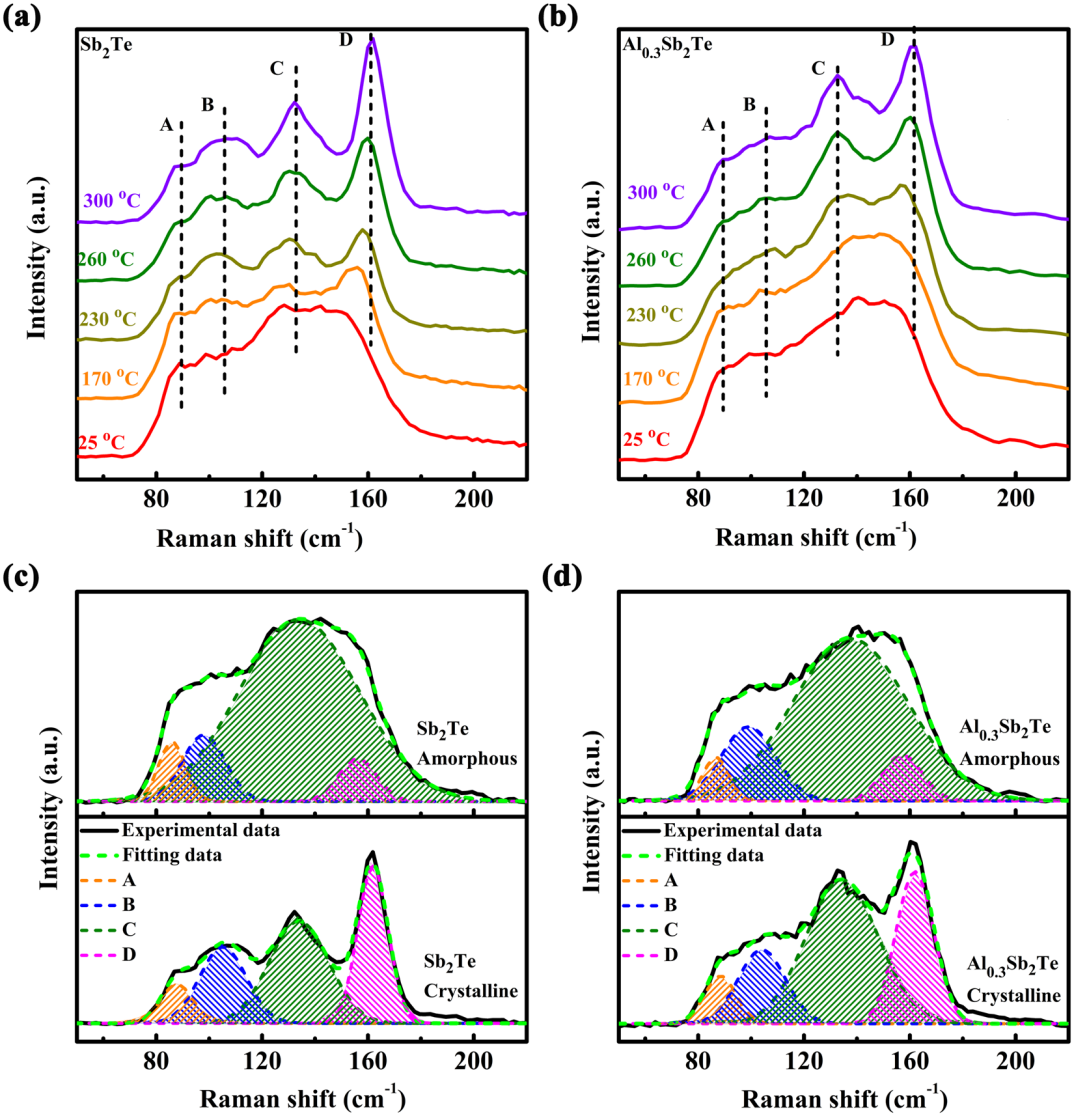
Temperature dependent Raman spectra of (**a**) ST and (**b**) AST films from 25 to 300 °C, respectively. Label C and D represent the two main Raman peaks of ST and SST films in hexagonal phase. Gaussian fitting of Raman spectra for crystalline and amorphous (**c**) ST and (**d**) AST films.

Raman spectroscopy is employed to characterize the binding environment in ST and AST through identifying the unique vibration modes of bonds between Al, Sb, and Te atoms, presented in Fig. 3. The curves of amorphous ST and AST are quite similar, as can be seen from Fig. 3(a,b), that contain a broadening band covering the frequency from 60 to 180 cm^−1^, originated from the complex binding environment in the amorphous atoms metrics. After crystallization, atoms arrangement shows long range order, resulting in centralized distribution of vibration modes at four frequencies, marked as A, B, C, and D. To better distinguish the different vibration modes, Gaussian line-fitting has been applied on the Raman results, as shown in Fig. 3(c,d). Goodness (R^2^) of fits are ~ 0.99, close to the perfect fit value of 1, showing that the fitting lines are quite close to the experimental curves. Detailed parameters of each peak are listed in Table 1. The peak C at ~135 cm^−1^ and peak D at ~162 cm^−1^ attribute to ${A}_{{\rm{1g}}}$ mode of homopolar Sb-Sb bonds^28^ and ${A}_{{\rm{1g}}}^{2}$ mode in Sb_2_Te_3_^29^ quintuple layers, respectively, which are the two basic components in the 9-layers ST unit of Sb_4_-(Sb_2_Te_3_)^30^. The peak A at ~90 cm^−1^ and peak B at ~105 cm^−1^ result from ${E}_{g}$ mode of homopolar Te-Te bonds^31^ and ${E}_{g}$ mode of homopolar Sb-Sb bonds^19^, respectively.

**Table 1:** Raman spectra peak identity of AST and ST (in parentheses).

Peak identity	Amorphous		Crystalline	
	Intensity (arb. units)	Wavenumber (cm^−1^)	Intensity (arb. units)	Wavenumber (cm^−1^)
A	3 (4)	87 (86)	3 (2)	88 (87)
B	11 (8)	99 (98)	9 (10)	104 (105)
C	50 (55)	137 (134)	27 (16)	135 (135)
D	5 (5)	157 (156)	14 (13)	162 (162)

Higher intensity of a peak is associated with high proportion of motifs possessing that vibration mode. From Fig. 3(c,d), it can be inferred that Sb-Sb units prevail in amorphous ST and AST, as peak C shows higher intensity than the other ones. Better crystallization has been achieved when annealed under higher temperature, leading to continuous formation of Sb_4_-(Sb_2_Te_3_) units in both ST and AST and sequential amplification of peak D, as displayed in Fig. 3(a,b), respectively. From the curves of 300 °C annealed ST and AST, one will notice that the intensity ratio of peak D vs. peak C (I_D_/I_C_) in AST is lower than that of ST, indicating that the portion of Sb_2_Te_3_ quintuple layers in AST is less than that in ST. That can be explained by the substitution of Sb by Al in Sb_2_Te_3_ part of Sb_4_-(Sb_2_Te_3_) unit, making the vibration mode differ from ${A}_{{\rm{1g}}}^{2}$ mode of Sb_2_Te_3_, thus decreasing its contribution to peak D.

**Figure 4:**
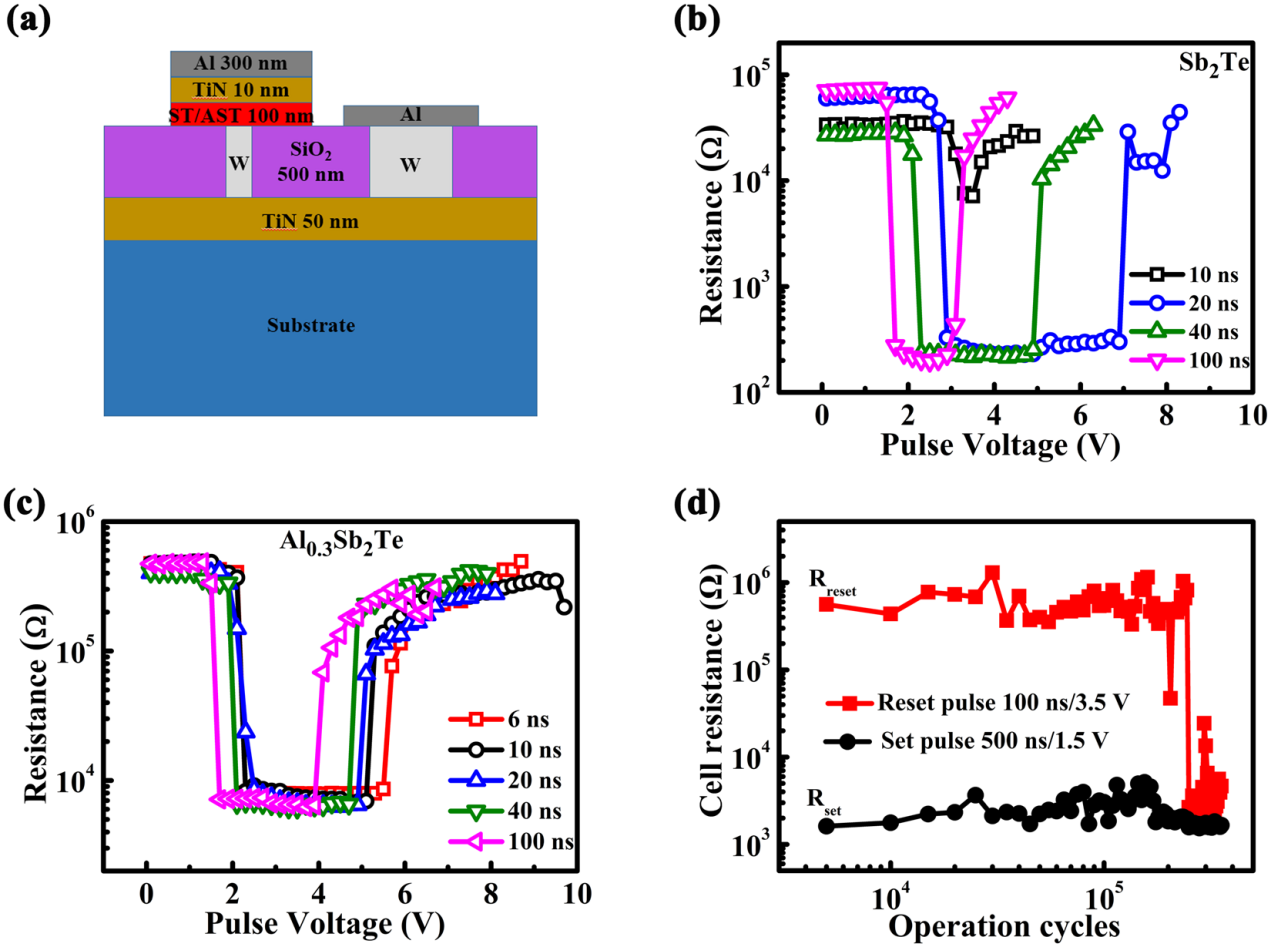
(**a**) Schematic diagram of the cross section of the PCM cell. (**b**,**c**) Resistance-voltage characteristics of PCM cells based on ST and AST with different voltage pulse widths. (**d**) Endurance characteristic of AST based PCM cell.

The electrical triggered phase change ability of ST and AST is characterized in T shaped PCM cells, the structure of which is illustrated in Fig. 4(a). The Set and Reset operations of the PCM cells are realized by voltage pulses applied across the cell, as presented in Fig. 4(b,c) for ST and AST, respectively. The sensing margin of AST based cell is ~2 orders of magnitude (Reset resistance ~10^5^Ω, Set resistance ~10^3^Ω), equal to that of AST based cells (Reset resistance ~10^4^Ω, Set resistance ~10^2^Ω). High resistance of AST based cell in Set state is regarded as a benefit of lowering the driven current, improving the Joule heat efficiency^32^. Furthermore, the Set speed has been significantly improved after Al doping, for a Set operation has been achieved by 6 ns pulse in AST based cell, while it should be ~20 ns in ST based cell. To make the 6-ns Set/Reset operation more convincing, the resistance-voltage characteristics of 10 different cells are tested under 6 ns pulse width as shown in Fig. S1. The 6-ns Set/Reset speed has preceded the 10-ns speed for DRAM, making AST a promising candidate for high-speed DRAM-like PCM application. The endurance of AST based cell is over 2 × 10^5^ under 500 ns/1.5 V Set pulse and 100 ns/3.5 V Reset pulse, displayed in Fig. 4(d). More cycling data could be found in Fig. S2 and the possible failure mechanism is discussed in the supplementary file.

## Discussion

**Figure 5:**
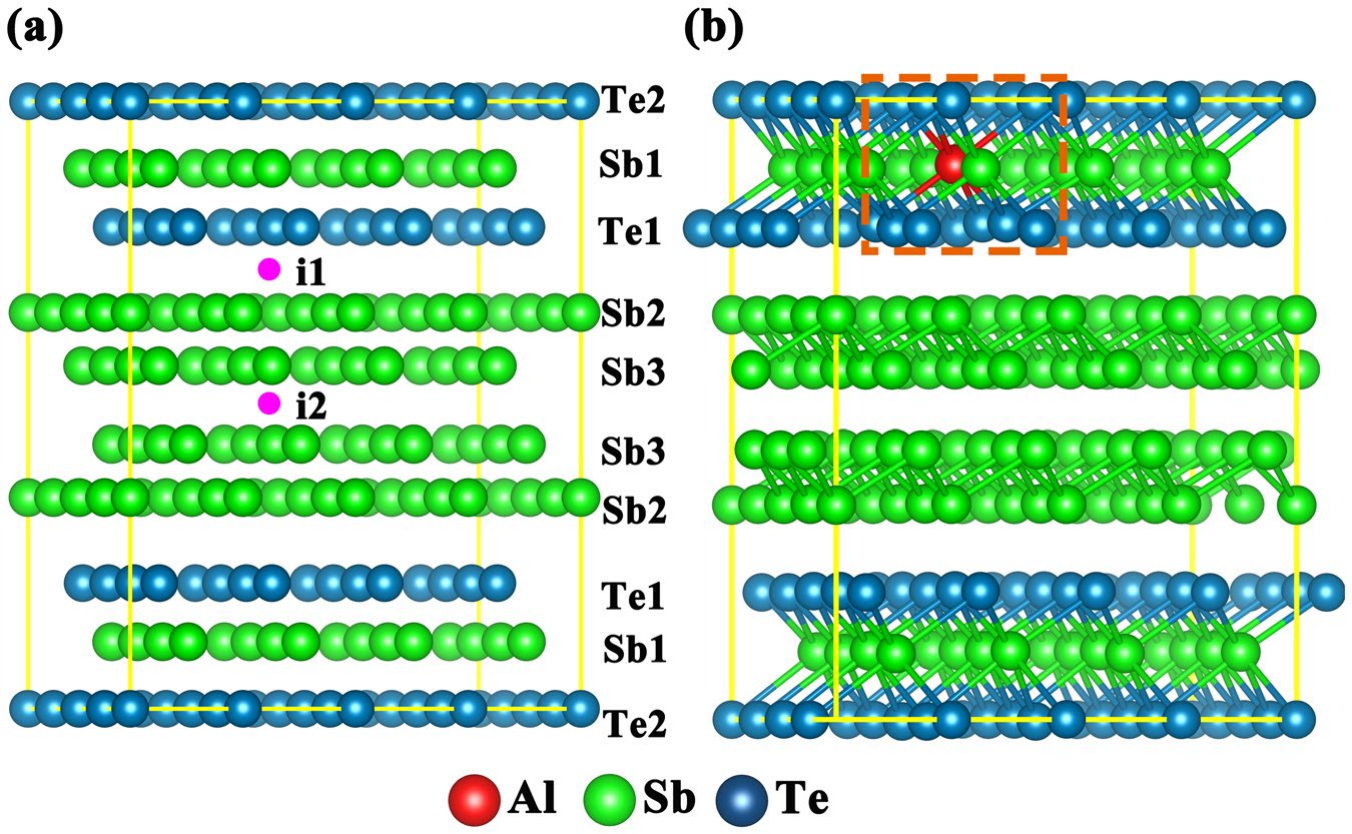
The seven possible doping types and the structure of Al doped crystalline Sb_2_Te. (**a**) Crystal structure of the 4 × 4 × 1 supercell of Sb_2_Te, each slab formed by 9 layers stacked along c in the sequence -Te1-Sb1-Te2-Sb1-Te1-Sb2-Sb3-Sb3-Sb2-, of which the Te atoms where weak van der Waals force exists are defined as Te1. There are five possible substitutional sites at Sb1, Sb2, Sb3, Te1, and Te2, as well as one interstitial site i1 between the adjacent Te1 and Sb2 layers and another interstitial site i2 between the adjacent Sb3 layers for the dopant Al. (**b**) Sixfold coordinated Al atoms in the hexagonal structure. It is obvious that Al atoms are in a (defective) octahedral like environment.

The preference position of Al in crystalline ST (c-ST) is studied using *ab-initio* method, through the calculation of the formation energy (*E*_*f*_) of each possible site, as shown in Fig. 5. A simple system, containing only one Al atom in one Sb_4_-(Sb_2_Te_3_) unit, has been used to lower the complexity of the analysis. Seven sites have been considered as the possible positions of Al atom, as shown in Fig. 5(a). Those are, substituting for the Te atom locating at the outmost layer of the Sb_2_Te_3_ quintuple layers (denoted as A_Te1_), substituting for the Te atom in the central layer of Sb_2_Te_3_ quintuple layers (denoted as A_Te2_), substituting for the Sb atom inside the Sb_2_Te_3_ quintuple layers (denoted as As_b1_), substituting for the Sb atom at the outmost layer of the Sb_4_ quadruple layers (denoted as A_Sb2_), substituting for the Sb atom inside the Sb_4_ quadruple layers (denoted as A_Sb3_), occupying an interstice site between the neighboring quintuple layers and Sb layers (denoted as A_i1_) and occupying the interstice side between the neighboring Sb bi-layers (denoted as A_i2_). A_i1_ and A_i2_ are projected at the geometric center of the (001) plane. The *E*_*f*_ of forming those Al defects is calculated as follows^33^:

2 $${E}_{f}={{E}}_{{tot}}[{AlS}{{b}}_{{2}}{Te}]-{E}_{{tot}}[S{b}_{{2}}{Te}]-{\mu }_{{Al}}+{\mu }_{{0}}$$

where ${E}_{{\rm{tot}}}[{AlS}{{b}}_{{2}}{Te}]$ and ${E}_{{tot}}[S{b}_{{2}}{Te}]$ are the total energy per supercell with and without Al dopants, respectively, ${\mu }_{{Al}}$ is the chemical potential of the dopant Al, and ${\mu }_{{0}}$ indicates the chemical potential of Sb and Te, respectively, while it is zero for A_i1_ and A_i2_. The chemical potentials for Al, Sb, and Te atoms are set to their total energies in the most stable crystalline structure. The calculated formation energies are shown in Table 2, among which the ${E}_{f}$ of A_Sb1_ is the lowest, indicating us the energy favorable location of Al atoms in AST, as presented by red ball in Fig. 5(b). In the octahedral-like environment as A_Sb1_, Al atoms are confirmed to be sixfold coordinated.

**Table 2:** Formation energy of different doping site.

Doping site	A_Te1_	A_Te2_	A_Sb1_	A_Sb2_	A_Sb3_	A_i1_	A_i2_
Formation energy (eV)	0.3814	0.1855	−0.5561	0.2727	0.2424	1.0479	1.2455

**Figure 6:**
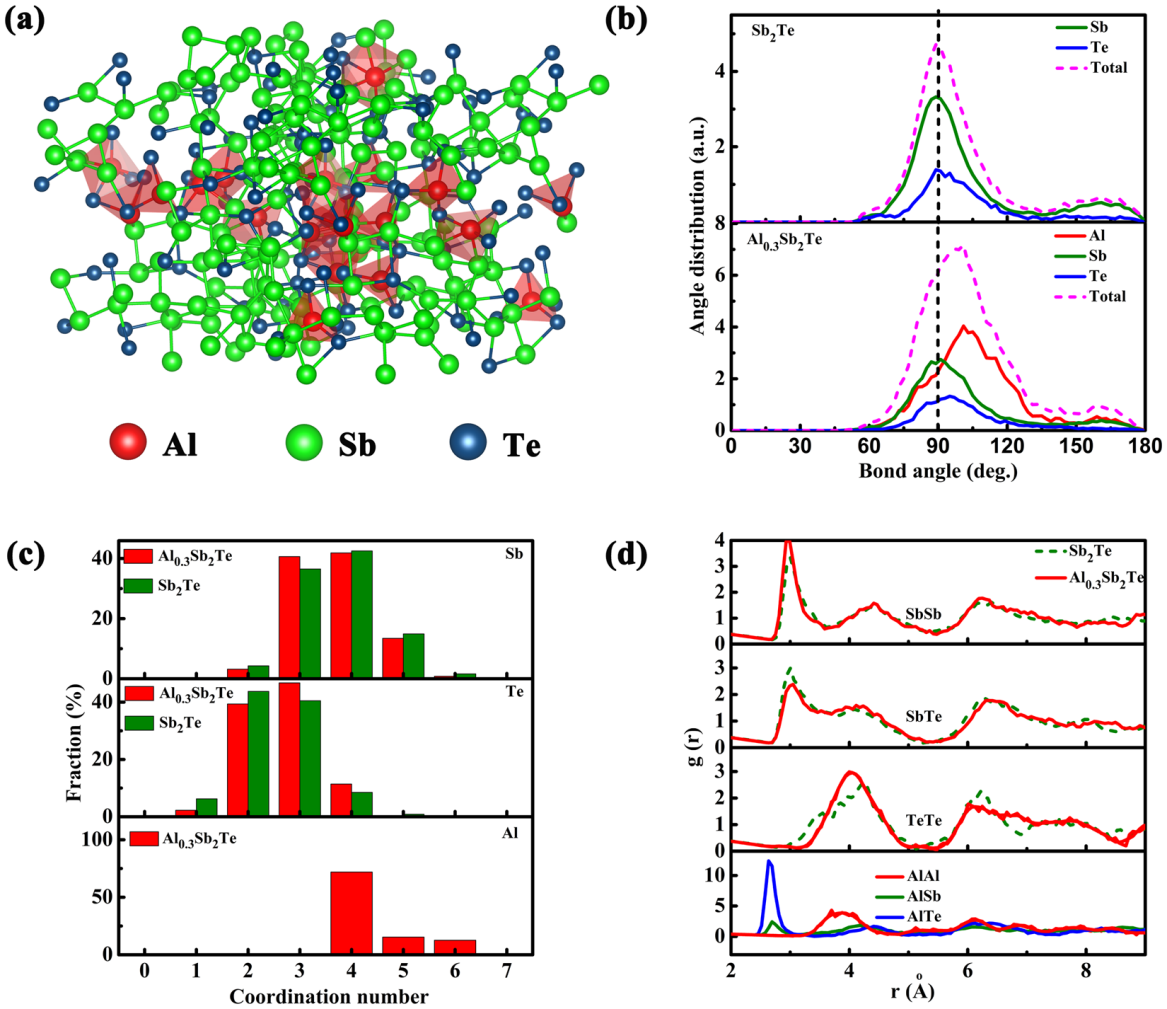
(**a**) A snapshot structure of amorphous AST. The amorphous structure of AST depicts that a majority of the Al are bonded with Te and fourfold coordinated in the tetrahedral-like geometry, which are highlighted as red polyhedrons. (**b**) Angle distribution functions of amorphous ST and AST. (**c**) Distributions of coordination numbers in amorphous ST and AST. (**d**) The partial paircorrelation functions of amorphous ST and AST at 300 K.

*Ab-initio* molecular dynamics (AIMD) simulations are performed to study the structural properties of amorphous ST (a-ST) and AST (a-AST). Here, how the Al impurities affect the short-range order of atoms arrangement in the amorphous phase has been discussed. Figure 6(a) gives a snapshot of atoms in a-AST, in which the Al-centered tetrahedrons (mostly Al-Te4) prevail, highlighted in red. One can also find some Al-Sb “wrong bonds” in Sb-Al-Te3 tetrahedrons, which account for the enhancement of amorphous thermal stability. The bond-angle distribution functions are calculated for a-ST and a-AST, where the cutoff radius is 3.2 Å for all atomic pairs, displayed in Fig. 6(b). The bond angles ~90° are dominant around Sb and Te atoms, in both a-ST and a-AST, indicating that most of Sb and Te atoms are in the defective octahedral-like environment. Whereas, the bond angle around Al atoms is ~109°, showing a defective tetrahedral-like environment, resemble to the tetrahedral Ge centered motifs in amorphous Ge_2_Sb_2_Te_5_^34,35^.

The distribution of coordination numbers for Al, Sb, and Te atoms in a-ST and a-AST are counted, exhibited in Fig. 6(c), with the average coordination numbers listed in Table 3. The cutoff radius that used to compute coordination numbers is set to be 3.2 Å for all atomic pairs. In both a-ST and a-AST, most of the Sb atoms are threefold and fourfold coordinated. Te atoms are largely threefold and fourfold coordinated. While, Al atoms are with high coordination numbers, e.g. fourfold, fivefold, and sixfold, among which fourfold is predominant. The much difference between the fourfold coordinated Al in a-AST and sixfold coordinated Al in c-AST has provided a strong hint for one to conclude that Al atoms undergo tetrahedron-octahedron rearrangements during phase transition of AST. To finish the transition of AST from amorphous to crystalline, the octahedral Sb atoms need to align near the matrix boundary, whereas the Al-Sb “wrong bonds” may delay the motion of Sb atoms, attributed to higher activation energy of crystallization and a better thermal stability of the amorphous phase.

**Table 3:** Average coordination numbers of Al, Sb and Te atoms in amorphous Al_0.3_Sb_2_Te and (in parentheses) in Sb_2_Te.

	Total	With Sb	With Te	With Al
Sb	3.693 (3.624)	2.489 (2.408)	1.020 (1.216)	0.184
Te	2.693 (2.537)	1.764 (2.433)	0.056 (0.104)	0.873
Al	4.409	1.178	3.222	0.009

The partial pair-correlation functions (PPCFs), extracted from the MD simulation at 300 K (red curve denotes a-AST and green dashed denotes a-ST), can provide more statistical information of the bonds in the amorphous states, as shown in Fig. 6(d). The cutoff radius is 3.2 Å for all atomic pairs. For PPCFs of a-AST, the higher peak of Sb-Sb at ~3.0 Å and lower peak of Sb-Te at ~3.0 Å, compared to that of a-ST, indicate an increase of Sb-Sb homopolar bonds and a decrease of Sb-Te bonds. That is, originated from the favorable binding between the more metallic Al (electron negativity: Al 1.61, Sb 1.98) and nonmetallic Te, as proved by the intense Al-Te peak at ~2.70 Å. Consequently, the leftover Sb atoms need to form homopolar Sb-Sb bonds, causing an increase in Sb-Sb bonds. As both ST and AST are Sb-rich material, Te atoms are depleted when binding with Sb and Al atoms. Thus, there is no Te-Te bonds in a-AST, reflected on the distance between the most neighboring Te atoms to be not less than 4 Å, presented in Fig. 6(d). The formation of short and strong bonds (Al-Te and Al-Sb) and the absence of weak Te-Te bond in a-AST ensures a high atomic network rigidity, in other words, good thermal stability.

## Conclusions

In summary, AST has been proposed to serve as storage media in PCM, that favors both a high writing speed (6 ns) and a good retention (103 °C), as well as a low power consumption. By Al doping, the *T*_*c*_ of AST has been increased to 200 °C, with the 10-year data retention temperature being enhanced to 103 °C. Confirmed by XRD and TEM, the grain size has been reduced by Al doping, while causing no segregation. Judging by Raman and *ab-initio* calculation results, doped Al atoms prefer to replace Sb in Sb_2_Te lattice, strongly bonded with 6 Te atoms, to form a homogeneous phase. While in amorphous Al doped Sb_2_Te (AST), Al atoms are in tetrahedral environment, firmly bonded with four Sb/Te atoms. The strong bonding in Al centered tetrahedron in amorphous AST can obstruct the collective motion of Sb atoms near the matrix boundary, leading to the improvement in thermal stability and the confinement in grain size.

## Experiments and Methods

### Sample preparation and experimental details

The ST and AST films were prepared by radio-frequency (RF) magnetron sputtering method at 21 °C under a base pressure of 2.0 × 10^−4^ Pa. The films for resistance-temperature (*R-T*), X-ray diffraction (XRD), and Raman tests were about 300 nm thick, deposited on the SiO_2_/Si (1 0 0) substrates. R-T was *in-situ* measured on a homemade hot stage with a heating rate of 20 °C/min. XRD measurements were performed by PANalytical X’Pert PRO diffractometer with Cu K_α_ (λ = 0.15418 nm) radiation source. Raman spectra are collected in HORIBA Jobin Yvon HR800 system at room temperature, using an Ar + laser (wavelength 514 nm) with ~1 µm^2^ beam spot.

T-shaped PCM cell fabricated by 0.13 μm CMOS technology was utilized to verify the electrically induced phase change ability of ST and AST. Phase change material layer (~100 nm), TiN layer (~10 nm) and Al top electrode (~300 nm) were sequentially deposited on the W bottom electrode, which is cylinder with a diameter of 190 nm. All the electrical measurements were performed by using the Keithley 2600 C source meter, the Tektronix AWG5002B pulse generator.

### *Ab-initio* theoretical simulation

In this work, *ab-initio* calculations based on the density functional theory (DFT)^36^ was employed. The Vienna *ab-initio* Simulations Package (VASP)^37^ was used for the first-principles calculations. The projector augmented wave (PAW)^38^ pseudopotentials were employed to describe electron-ion interactions, we used the generalized gradient approximation (GGA) based on the Perdew-Burke-Ernzerhof (PBE)^39^ function for the exchange-correlation energies between electrons. 7 × 7 × 2 was chosen as the *k* points for crystalline model which is built on the basis of a Sb_2_Te hexagonal cell with 9 atoms. It is well known that the choice of functionals in DFT calculations can affect the calculated force constants and hence the lattice significantly, especially with the existence of van der Waals (vdW) interactions in weakly bonded layered crystal structures, e.g. in Sb_2_Te_3_ and Sb_2_Te^40^. Among different functions, DFT-D2^41,42^ has been determined to be the most appropriate one, since it gives the best approach to the structure properties of ST, as shown in Table S1. So, a semiempirical dispersion potential was added to the conventional Kohn-Sham DFT energy in the scheme of the DFT-D2 method for the calculations of the Sb_2_Te structures. Based on the DFT-D2 calculations, the optimized lattice constants a and c for the rhombohedral structure of Sb_2_Te were 4.27 Å and 17.65 Å, respectively, in good agreement with experimental values^43^ and other theoretical calculations^44^. A 4 × 4 × 1 Sb_2_Te hexagonal supercell was used to calculate the formation energy. ST and AST were simulated with periodic boundary conditions by NVT molecular dynamics (MD): The ensemble of 144 atoms which was built on the basis of a 4 × 4 × 1 Sb_2_Te hexagonal supercell was melted and equilibrated at 3000 K for 9 ps, then quenched to 1200 K for another 30 ps, after equilibrated at 1200 K for 30 ps, then quenched to 300 K with a quench rate of −15 K ps^−1^ and finally maintained at 300 K for another 15 ps. The energy cutoff was chosen to be 300 eV for our models and a 3-fs time step was used for the MD.

## Electronic supplementary material

Supplementary Information
