# Supplementary material for: Atomic scale insight into the effects of Aluminum doped Sb2Te for phase change memory application
Source: Sci Rep. 2018 Oct 11;8:15136. doi: 10.1038/s41598-018-33421-y (PMC6181964; doi:10.1038/s41598-018-33421-y)
Supplement: Supplementary file 1 — Supplementary Information [file 41598_2018_33421_MOESM1_ESM.pdf]

Atomic scale insight into the effects of Aluminum doped Sb_2Te for phase change memory application

Yong Wang[1,3] , Tianbo wang[1,3], Yonghui Zheng[1,3], Guangyu Liu[1,3], Tao Li[1,3], Shilong Lv[1], Wenxiong Song[1], Sannian Song[1], Yan Cheng [1,4], Kun Ren[1,2,a)] & Zhitang Song[1]

[1]*State Key Laboratory of Functional Materials for Informatics, Laboratory of Nanotechnology, Shanghai Institute of Micro-System and Information Technology, Chinese Academy of Sciences, Shanghai 200050, China*

[2]*Hangzhou Dianzi Univ, Coll Mat & Environm Engn, Hangzhou, Zhejiang 310018, China*

[3]*University of Chinese Academy of Sciences, Beijing 100049, China*

[4]*Key Laboratory of Polar Materials and Devices, Ministry of Education, East China Normal University, Shanghai, 200062, China.*

*****Corresponding authors. E-mail address: kun.ren.nick@outlook.com

Supplementary Information

1. The choice of functionals in DFT calculations

In the hexagonal structure of Sb_2Te, the van der Waals (vdW) interactions cannot be ignored. Actually, LDA and GGA are not able to describe vdW interactions in weakly bonded layered crystal structures[1]. The choice of functionals in DFT calculations which can affect the calculated force constants and hence the lattice significantly and density becomes crucial. To better describe the vdW interactions in layered Sb_2Te, the functionals that approximately account for vdW interactions in DFT calculations including DFT-D2, optPBE-vdW, optB88-vdW, optB86b-vdW[2], and vdW-DF2, are tested as listed in Table S1.

Table S1 The effects of functionals on the structure and density.

	a / Å	c / Å	D / (Mg/m^{-3})
PBE	4.367	17.838	6.27
DFT-D2	4.277	17.654	6.60

optPBE-vdW	4.393	18.106	6.10
optB88-vdW	4.373	17.869	6.24
optB86b-vdW	4.351	17.479	6.45
vdW-DF2	4.510	19.286	5.44
Exp.[3]	4.272	17.633	6.63

DFT-D2 is found to be in good agreement with experimental values among these functionals. In order to consider the vdW interactions, so we choose the scheme of the DFT-D2 method to calculate the Sb_2Te structures in this work.

2. More test results on AST-based PCM cells

Figure S1 shows the resistance-voltage result of other cells in Al doped Sb_2Te (AST)-based PCM under 6 ns pulse width which can make 6-ns operating speed more convincing. The failure mode as shown in Figure S2(a) also belongs to the SET-stuck failure mode, and the failure models shown in Figures S2(b) and S2(c) belong to in-complete crystalline filament model[4]. It is speculated that these two failure models of AST may be caused by two main reasons: one is that the TiN material layer as the isolation adhesion layer and the AST layer of the phase change material interact with each other, and mutual diffusion between materials occurs; the second is the phase change material layer itself undergoes material migration changes, and the material may undergo vertical or horizontal diffusion migration under the action of electric field and thermal field.

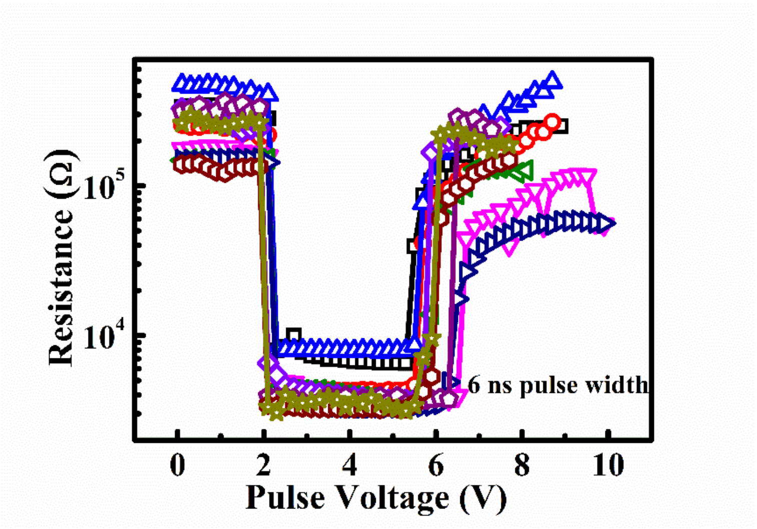

Figure S1 Resistance-voltage curves for other AST-based PCM cells under 6 ns pulse width

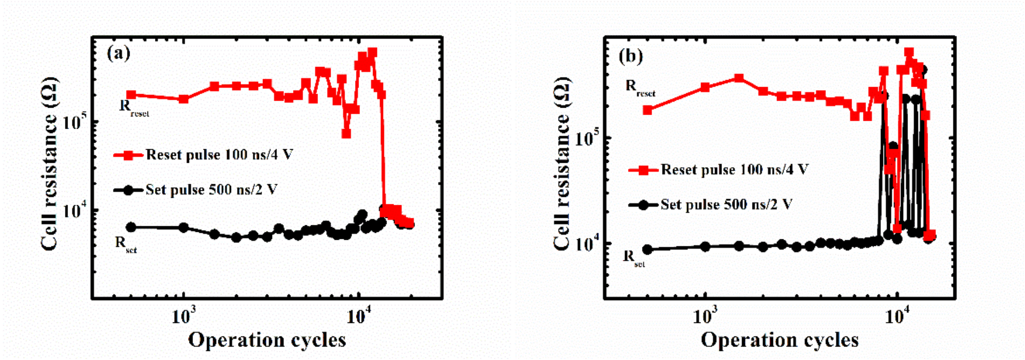

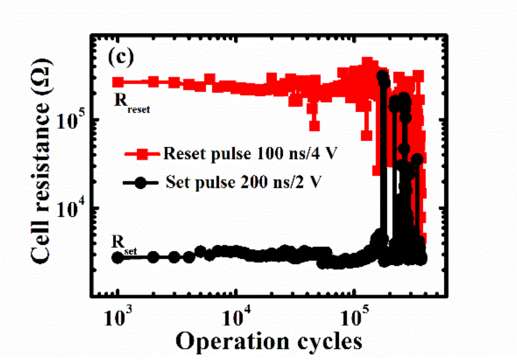

Figure S2 Endurance characteristics of other AST-based PCM cells

Supplementary Information References

1. Li, Z., Miao, N., Zhou, J., Xu, H. & Sun, Z. Reduction of thermal conductivity in $Y_xSb_{2-x}Te_3$ for phase change memory. *Journal of Applied Physics* **122**, 195107 (2017).
2. Klimeš, J., Bowler, D. R. & Michaelides, A. Van der Waals density functionals applied to solids. *Phys. Rev. B* **83**, 772-772 (2011).
3. Agafonov, V., *et al.* Structure of Sb_2Te, *Acta Crystallogr.* **47(6)**, 1141-1143 (2014).
4. Mantegazza, D., *et al*. Incomplete Filament Crystallization During Set Operation in PCM Cells. *IEEE Electron Device Letters*, **31(4)**, 341-343 (2010).
